# Effects of Preventive Exposure to High Doses of Alpha-Lipoic Acid (ALA) on Testicular and Sperm Alterations Caused by Scrotal Heat Shock in Mice

**DOI:** 10.3390/biology14121708

**Published:** 2025-11-30

**Authors:** Luciano Cardoso Santos, Maíra Guimarães Kersul, William Morais Machado, Jeane Martinha dos Anjos Cordeiro, Bianca Reis Santos, Cibele Luz Oliveira, Cleisla Souza Oliveira, Larissa Rodrigues Santana, Juneo Freitas Silva, Paola Pereira das Neves Snoeck

**Affiliations:** 1 Electronic Microscopy Center (CME), Department of Biological Sciences (DCB), State University of Santa Cruz (UESC), Ilheus 45662-900, Bahia, Brazil; jmacordeiro@uesc.br (J.M.d.A.C.); brsantos@uesc.br (B.R.S.); cloliveira.ppgca@uesc.br (C.L.O.); csoliveira.mev@uesc.br (C.S.O.); jfsilva@uesc.br (J.F.S.); 2 Laboratory of Biostatistics and Epidemiology of Parasitic Diseases, Department of Agricultural and Environmental Sciences (DCAA), State University of Santa Cruz (UESC), Ilheus 45662-900, Bahia, Brazil; mgkersul@uesc.br; 3 Department of Veterinary Medicine, Irecê College (FAI), Irecê 44900-000, Bahia, Brazil; william.morais@faifaculdade.com.br; 4 Animal Reproduction Laboratory (LARA), Department of Agricultural and Environmental Sciences (DCAA), State University of Santa Cruz (UESC), Ilheus 45662-900, Bahia, Brazil; lrsantana@uesc.br

**Keywords:** heat stress, thioctic acid, testicular thermoregulation, testis, mouse

## Abstract

Alpha-lipoic acid (ALA) is well-known for its antioxidant and anti-inflammatory functions in various disease models. Here, we tested whether pre-exposure to ALA can protect the testes from cellular damage caused by scrotal heat shock (HS) in mice. Methods: Thirty-six Swiss albino mice were divided into control (CTRL, n = 6), HS (n = 10), and two ALA dose (HS + ALA 200 mg/kg, n = 10; and HS + ALA 400 mg/kg, n = 10) groups. ALA supplementation was administered orally for 30 days. Subsequently, the animals, except the controls, were subjected to an HS water bath at 43 °C for 20 min. Two days later, they were euthanized, and biometric data from gonads and accessory sexual glands, testicular samples for histomorphometric and immunohistochemical analyses, and sperm from the epididymis cauda were obtained for evaluation. Results: Animals submitted to HS had a lower body weight, decreased relative mass of testes and prostate, reduced seminiferous epithelium height and tubular diameter, and increased degeneration in seminiferous tubules. Additionally, sperm analysis showed a reduced linear progressive velocity (VSL) and straightness (STR), increased midpiece defects, and fewer sperm with functional membranes. Immunohistochemical evaluation revealed a reduced superoxide dismutase (SOD1) staining intensity in the testes. Preventive exposure to ALA at 200 mg/kg did not normalize the relative testicular mass, but it reduced the number of giant cells, decreased midpiece defects, normalized the number of sperm with functional membranes, and partially preserved SOD1 expression. Although animals treated with ALA 400 mg/kg showed an improvement in relative testicular mass, this dose was less efficient in other parameters. Conclusions: This study showed that while 30 days of oral ingestion of ALA before the induction of acute degenerative injury did not fully protect male mouse gonads at the tissue level, some parameters related to testicular function and sperm quality showed a partial improvement.

## Simple Summary

This study investigated whether oral supplementation with alpha-lipoic acid (ALA), an antioxidant, can protect testicular tissue from damage caused by scrotal heat stress (HS). Swiss albino mice were divided into four groups: control, placebo, and two experimental groups receiving 200 mg/kg or 400 mg/kg of ALA daily for a month before being subjected to HS (43 °C scrotal immersion for 20 min). We measured the impact on testicular weight, accessory glands, sperm quality, and tissue structure. HS reduced testicular weight in all groups, while ALA supplementation had mixed effects on tissue health. Although higher ALA doses showed a partial improvement in certain parameters, significant damage to testicular tissue, sperm membrane integrity, and chromatin were still observed. Sperm movement and morphology did not differ between treatments. These findings suggest that high doses of ALA supplementation before HS do not provide complete protection for testicular health, highlighting the need for further research into effective strategies for preventing heat-related reproductive damage.

## 1. Introduction

As global temperatures continue to rise due to environmental changes, concerns regarding the impact of heat exposure on male reproductive health have gained prominence in both environmental and biomedical research. Recent global studies have documented a significant decline in sperm quality across populations over the past decades [1,2], raising questions about environmental and lifestyle factors contributing to male reproductive dysfunction. Although multifactorial, elevated scrotal temperature has emerged as a potential contributing factor, observed in both chronic exposures and acute thermal events.

Testicular thermoregulation is an essential physiological mechanism that maintains testicular functions, particularly gamete production, by ensuring that the testicular temperature remains lower than the body temperature [3,4]. Any intrinsic or extrinsic factor that causes scrotal hyperthermia or abnormal thermoregulation results in metabolic responses that generate Reactive Oxygen Species (ROS) [5]. Depending on the severity and frequency, this can lead to testicular degeneration [6,7,8].

In the testicular environment, the presence of natural antioxidants keeps ROS at an acceptable level [9], until injurious factors such as heat shock (HS) contribute to their imbalance and lead the testes into oxidative stress, possibly followed by apoptosis [5,10], thereby contributing to a lower sperm quality, and consequently, to male subfertility. Even a low HS is able to influence the homeostasis of testicular cells [11] because it can act as a catalyst for oxidative stress in the tissue [10,12,13] by inducing the upregulation of ROS and the downregulation of natural antioxidants [11].

To maintain or enhance these homeostatic control activities, antioxidant diet supplementation has become an investigated alternative due to its role in defending against ROS or its function in supplying essential cofactors for enzymatic antioxidant systems, such as superoxide dismutase (SOD), catalase (CAT), and glutathione peroxidase (GPx) [14]. Alpha-lipoic acid (ALA) and its reductive form, dihydrolipoic acid (DHLA), are considered universal antioxidants as they are both hydrosoluble and liposoluble, demonstrating effectiveness while inhibiting ROS in extra- and intracellular settings for oxidative stress prevention, and an ability to act as a coenzyme for enzymatic and non-enzymatic physiological antioxidant processes [15,16]. Nonetheless, in studies from Moini et al. [17] and Liu et al. [18], it was noted that ALA can also act as a coenzyme in mitochondrial metabolism, suggesting its potential to delay, prevent, or repair mitochondrial deterioration. In this sense, ALA is a potent antioxidant that has yielded encouraging results in several diseases that involve oxidative stress, such as in the kidneys [19,20], liver [21,22], heart [23,24], instances of traumatic brain injury [25], lungs [26,27], and other studies.

In male reproductive studies, increasing evidence has demonstrated that ALA can attenuate Sertoli cells’ loss, enhance sperm quality, and minimize damage in spermatic DNA [28,29], even in an induced varicocele [30], as well as decrease cell damage and apoptosis in testicular ischemia–reperfusion in rats by preventively controlling oxidative stress [31]. Other studies have also shown protective effects of ALA via antioxidant defense in testicular injury induced by methotrexate [32], doxorubicin [33], lipopolysaccharide (LPS) [34], and carbimazole [35]. When associated with ascorbic acid and tocopherol, ALA attenuates testicular oxidative damage induced by hyperglycemia and acts as a protector against germinative cells’ apoptosis [36]. Furthermore, when associated with other nutraceuticals, as described by Canepa et al. [37], improvements in sperm parameters for subfertile men were observed. Under heat stress conditions, on the other hand, only one study in chickens showed effects of ALA on oxidative damage and testicular endoplasmic reticulum stress [38].

In this exploratory study, we investigated whether ALA supplementation before HS could prevent or protect adult mice testes against sperm cell degeneration, as well as improve semen quality.

## 2. Material and Methods

### 2.1. Experimental Design and Animals

Thirty-six Swiss albino mice (*Mus musculus*; 8 weeks old; 35–40 g) were obtained from the Laboratory of Animal Breeding, Maintenance, and Experimentation (LaBIO) of the Santa Cruz State University (UESC) and housed under a controlled temperature (22 °C) and photoperiod (12 h of light and 12 h of darkness), with access to water and rodent chow ad libitum. The animals were randomly assigned to the following groups: control (CTRL; n = 6); scrotal heat shock + placebo (HS + P; n = 10); scrotal heat shock + 200 mg/kg ALA (HS + A200; n = 10); and HS + 400 mg/kg ALA (HS + A400; n = 10). The doses of 200 and 400 mg/kg were determined based on the study of Yusha’u et al. [39]. All groups, except the CTRL group, received oral supplementation for 30 days and were then subjected to acute testicular degeneration by an HS bath on the last day of treatment. The 30-day duration was chosen to simulate a realistic preventive intervention and to allow sufficient time for alpha-lipoic acid to exert systemic antioxidant effects, as supported by previous murine studies employing similar or longer administration periods [39,40,41,42,43]. Eight days before the euthanasia, all groups were separated into individual boxes with a female in estrus to deplete their extragonadal sperm reserves. Then, 48 h after the HS bath, the mice were sacrificed, and their biometric data and gonadal and extragonadal organs were collected for further studies. This study followed the Animal Research: Reporting of In Vivo Experiments (ARRIVE) guidelines (https://arriveguidelines.org/ accessed on 2 July 2025) and was approved by the Ethics Committee on Animal Use (CEUA) of UESC under protocol number 030/19.

### 2.2. ALA Administration and Acute Testicular Degeneration by HS

The ALA powder was purchased from ViaPharma^®^ (206, 33 g/mol, Itabuna, BA, Brazil) and stored in a refrigerator at 4 to 6 °C until its dilution in corn oil as a vehicle [44]. Acute testicular degeneration was induced by HS following the protocol proposed by Yon et al. [45]. In short, each mouse was sedated with 5 mg/kg intraperitoneal (I.P.) midazolam (Hipolabor Pharma LTDA, Belo Horizonte, Brazil). Then, its distal third of the body (including scrotum) was immersed for 20 min in a 43 °C water bath. After immersion, animals were dried with a surgical compress and placed under an incandescent lamp protected from audio-visual stimulus until we observed normal behavior and returned the animal to its cage.

### 2.3. Genital Tract Collection and Gonadosomatic/Glandular Index

The scrotum was opened and the testes removed and weighed. Based on the body and both testicular weights, the relative testicular mass was calculated by dividing the testicular weight by the body weight and multiplied by 100. Additionally, the vesicular glands and prostate were removed and weighed, and their respective weights were divided by the body weight to obtain the relative mass. To recover sperm, the epididymis was excised in 0.9% saline solution at 37 °C.

### 2.4. Sperm Collection and Analysis

The left epididymis was placed in a Petri plate warmed at 37 °C and carefully minced with a scalpel. It was then transferred to a microtubule with 250 µL of saline solution at 37 °C. After 15 min, the fluid was diluted in another 250 µL of saline solution at 37 °C for a further 15 min. The fluid was thoroughly mixed, and aliquots were collected for motility, sperm morphology, a hypo-osmotic test (HOST), and spermatic chromatin integrity analysis.

#### 2.4.1. Sperm Kinematics

Sperm motility was evaluated after re-diluting the semen with an additional 250 μL of physiological saline at 37 °C. The sample was incubated in a dry bath for 10 min, after which 3 μL was deposited by capillarity onto a Leja^®^ 20 slide and analyzed using a computerized system. The parameters used in the Sperm Class Analyser^®^ (SCA—Microptics S.L, Evolution, Veterinary Edition, Barcelona, Spain) for rodents included 25 images per second at 25 Hz, with particle sizes captured ranging from 5 to 70 μm/m^2^. Spermatozoa were classified as immotile (<10 μm/s), slow–medium (15 μm/s), and fast (>25 μm/s). The following parameters were evaluated: total motility (TM) and progressive motility (PM), expressed as a percentage; curvilinear velocity (VCL), linear progressive velocity (VSL), and average path velocity (VAP), expressed in micrometers per second (μm/s); linearity (LIN) and straightness (STR), expressed as a percentage; and amplitude of lateral head displacement (ALH), expressed in micrometers (μm).

#### 2.4.2. Sperm Concentration and Morphology

The concentration was evaluated using a Neubauer chamber after fixing 10 μL of semen in 190 μL of 4% formalized sodium citrate solution (dilution ratio 1:20). The chamber preparation and sperm counting followed the recommendations of the Manual for Andrological Examination and Animal Semen Evaluation [46].

For sperm morphology assessment, the same fixed sample used for concentration analysis was utilized, and the studies by Takeda et al. [47], Musa et al. [48], and Skinner et al. [49] provided insights into expected pathologies in mice. The wet preparation between slide and coverslip followed the Manual for Andrological Examination and Animal Semen Evaluation [46]. Morphological alterations were evaluated using a contrast microscope at 1000× magnification (Olympus^®^ CX31 Tokyo, Japan) with immersion oil, and at least 200 cells were analyzed, with us categorizing alterations, when present, according to the classification by Rao [50].

#### 2.4.3. Hypoosmotic Test (HOST)

The functional integrity of the plasma membrane was assessed by diluting 10 μL of semen in 40 μL of distilled water (dilution ratio 1:5), followed by incubation in a dry bath at 37 °C for 15 min. The percentage of reactive sperm was then evaluated on a slide under a coverslip, according to the wet preparation technique [46]. A total of 100 spermatozoa were analyzed using a phase contrast microscope (Olympus^®^ CX31) at 1000× magnification with immersion oil. The reactive percentage value was adjusted by subtracting the percentage of spermatozoa with coiled or bent tails observed during the morphological assessment.

#### 2.4.4. Structural Integrity of Membranes

For the analysis of the structural integrity of the plasma and acrosomal membranes, two fluorochromes were used: carboxyfluorescein diacetate (CFDA) and propidium iodide (PI). A 10 μL aliquot of semen was incubated in 40 μL of working solution containing fluorescent probes, following the protocol of Harrisson and Vickers [51]. The sample remained at room temperature for eight minutes before being analyzed via fluorescence microscopy (Olympus^®^ BX51 Tokyo, Japan) at 400× magnification. CFDA staining was evaluated using the standard fluorescein filter set, while PI staining was assessed using the standard rhodamine filter set. A total of 100 spermatozoa were analyzed and classified into three categories: intact (CFDA+/PI−), damaged (CFDA−/PI+), and partially damaged (CFDA+/PI+). For the analysis of treatment effects, only intact spermatozoa were considered.

#### 2.4.5. Chromatin Assessment Test

The integrity of sperm chromatin was assessed using the metachromasia technique induced by toluidine blue [52]. Smears were prepared with a 10 μL aliquot of the sample, dried at room temperature, and fixed for 1 min in Carnoy’s solution (3:1, 75 mL of 100% alcohol + 25 mL of acetic acid), followed by fixation in 70% alcohol for three minutes. Hydrolysis with 4N hydrochloric acid was performed for 15 min, followed by washing in distilled water and drying at room temperature. For smear staining, 10 μL of 0.025% toluidine blue solution (0.00125 g of toluidine blue in 5 mL of McIlveine solution, pH 4.0) was deposited between the slide and coverslip. A total of 200 cells were analyzed using phase contrast microscopy (1000×; Olympus^®^ CX31). Spermatozoa were classified as having compact chromatin (head region stained light blue) or uncompacted chromatin (head region stained dark blue or violet).

### 2.5. Testicular Histomorphometry and Histopathology

Testes were fixed in paraformaldehyde 4% for 24 h, followed by alcohol dehydration and paraffin embedding. Then, 4 µm thick sections were stained with Hematoxylin and Eosin (H&E) staining. Micrographs were captured using light microscopy (Leica Microsystems. Inc., DM2500, Wetzlar, Germany, 200× and 400×).

The volumetric density of all seminiferous tubule components was determined after placing a 108-intersection grid from WCIF ImageJ^®^ software version 1.41 (Media Cybernetics Manufacturing, Rockville, MD, USA) on ten randomly selected regions per testis of each animal, examined with 400× magnification (1080 points/animal). For testicular biometry, approximately 30 round tubules were randomly chosen for tubular measurements also using ImageJ. The tubular diameter was obtained by averaging two cross-sections from each tubule from each individual, and the epithelium height from the same tubular sections was determined using an average of four measurements of opposite poles. To obtain the area measurement from tubular, luminal, and epithelial compartments, we applied the formulas described by Dias et al. [53] with the values from the previously described method.

For histopathological study, we counted 200 random testicular tubules from each individual and classified them proportionally by morphological alterations, as proposed by Johnsen [54] and adapted by Dias et al. [53], considering (a) mild pathologies—tubules with vacuoles at the base or apex of the epithelium (levels 2 and 3); (b) moderate pathologies—presence of vacuoles at the apex and base of the epithelium or spermatogenic cells in the tubular lumen (such as multinucleated giant cells) and cellular degeneration (levels 4 and 5); and (c) severe pathologies—tubules with only basal cells, only Sertoli cells, or without Sertoli or germ cells (levels 6, 7, and 8).

### 2.6. Radioimmunoassay (RIA) for Testosterone

Blood samples collected were centrifuged (Heraeus Megafuge 16R, Thermo Fischer Scientific^®^, Waltham, MA, EUA) at 1200× *g* for 20 min at 4 °C. The serum recovered with the aid of a pipette was stored in a microtube in the freezer at −20 °C until the time of evaluation using the Testosterone Radioimmunoassay (RIA) kit of the Immuchen Corp., formerly ICN Biomedicals Inc., (MP Biomedicals, Costa Mesa, CA, EUA), following the manufacturer’s instructions.

### 2.7. Immunohistochemistry (IHC)

For IHC, the Dako detection system (EnVision FLEX+, Mouse, High pH, (Link); Dako North America, Inc., Carpinteria, CA, USA), following the protocol of Ilie et al. [55], was used with some adaptations for mouse tissue. Briefly, the sections were deparaffinized in an oven (58–65 °C), diaphanized in xylene, and rehydrated in graded baths of ethyl alcohol. Antigen retrieval was performed by heating in citric acid buffer (0.54 mol/L; 98 °C; pH 6.0) for a maximum time of 40 min. Peroxidase blocking was performed in hydrogen peroxide solution (3%; H_2_O_2_) diluted in methanol (CH_3_OH) for 30 min. The tissue sections were incubated with bovine serum (5%) to block nonspecific binding for 30 min, and then incubated for 1.5 h in IgG blocking solution (ReadyProbes™ Mouse-on-Mouse; no. R37621; Life Technologies Corporation, Carlsbad, CA, USA). The sections were incubated overnight with anti-SOD1 (1:500, sc-101523, Santa Cruz Biotechnology, Paso Robles, CA, USA). Subsequently, a protein stabilization solution (EnVision™ FLEX+, Dako North America, Inc., Carpinteria, CA, USA) was added for 30 min, followed by the addition of secondary antibody conjugated to streptavidin peroxidase (EnVision™ FLEX/HRP, Dako North America, Inc., Carpinteria, CA, USA) for another 30 min. The chromogen used was 3′3 diaminobenzidine (EnVision™ FLEX DAB+ Chromogen, Dako North America, Inc., Carpinteria, CA, USA), diluted in buffer with H_2_O_2_ (EnVision™FLEX Substrate Buffer, Dako North America, Inc., Carpinteria, CA, USA, 1:50). Between each step, the tissues were washed with PBS. The sections were counterstained with Harris hematoxylin, and the negative control was obtained by replacing the primary antibody with PBS. At least 10 random fields were photographed per animal using a Leica DM 2500 microscope and a Leica DFC 295 digital camera (Leica Microsystems, Wetzlar, Germany) at 400× magnification. These photographs were analyzed by two blinded evaluators, and the parameters of (1) seminiferous epithelium, (2) interstitial cells, (3) giant cells (when present), and (4) apoptotic/injured cells (when present) were categorized according to a scale of staining intensity. The scale ranged from 0 to 4, with 0 = absent; 1 = weak; 2 = moderate; 3 = intense; and 4 = very intense. An average score was determined for each parameter studied.

### 2.8. Statistical Analysis

Normality of the distribution was tested by the Shapiro-Wilk normality test. Parametric data were subjected to analysis of variance (ANOVA) followed by the Bonferroni post hoc test. Nonparametric data were subjected to the Kruskal–Wallis and Dunn multiple comparison tests. Both were run in GraphPad Prism 9.5.0 (730)^®^. Comparisons between two groups were made using Student’s *t*-test. The results were considered statistically significant when *p* < 0.05, and they were presented as a mean ± standard error of the mean (SEM).

## 3. Results

### 3.1. Survival Rate After Scrotal Heat Shock (HS) Bath

**Figure 1:**
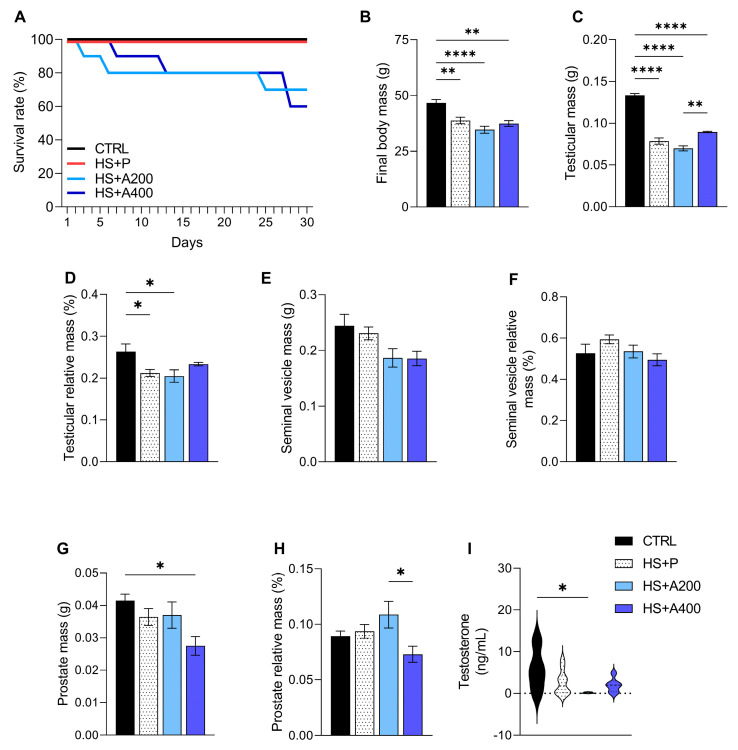
Biometric data of adult Swiss mice treated with alpha-lipoic acid (ALA) 30 days before acute testicular degeneration by heat shock (HS) (mean ± SEM). (**A**) Survival rate after initiation of oral exposure to ALA. (**B**) Body mass on the day of euthanasia. (**C**) Absolute testicular mass. (**D**) Relative testicular mass. (**E**) Absolute seminal vesicle mass. (**F**) Relative seminal vesicle mass. (**G**) Absolute prostate mass. (**H**) Relative prostate mass. (**I**) Serum testosterone values. Legend: CTRL—control without heat shock (n = 6); HS + P—scrotal heat shock-receiving placebo (n = 10); HS + A200 (n = 7) and HS + A400 (n = 6)—scrotal heat shock and daily doses of ALA; * *p* < 0.05; ** *p* < 0.01; **** *p* < 0.0001.

Survival rates were monitored daily for 30 days across all experimental groups. The CTRL group, not subjected to scrotal HS, maintained 100% survival throughout the study period. Similarly, the HS + P group also exhibited 100% survival, with no short-term mortality directly attributable to the HS protocol. In contrast, the administration of ALA at the tested doses affected survival. The HS + A200 group presented an initial survival rate of 100% but this declined to 80% by day 5, further decreasing to 70% by day 30. The HS + A400 group experienced a more rapid decline, reaching 80% survival by day 5 and concluding the study with 60% survival by day 30 (Figure 1A). Additionally, mild adverse effects were observed in ALA-treated animals, including episodes suggestive of gastroesophageal reflux, rare occurrences of diarrhea, and minor hair loss.

### 3.2. Body Mass, Testosterone, and Genital Tract Mass

First, we investigated the effect of HS on the morphometric characteristics of testes and accessory glands in mice. All groups subjected to HS showed a reduction in both body mass (Figure 1B) and absolute testicular mass (Figure 1C). Regarding testicular relative mass, the HS + A400 group demonstrated values similar to those of the CTRL group (Figure 1D); however, this parameter was lower in animals from the HS + P and HS + A200 groups (Figure 1D). No significant differences were observed in either the absolute or relative mass of the seminal vesicles among any of the treatments (Figure 1E,F). Conversely, both the absolute (Figure 1G) and relative (Figure 1H) prostate masses were lower in animals treated with HS + 400 compared to the CTRL and HS + A200 groups, respectively. As for testosterone levels, only animals treated with the 200 mg/kg dose of ALA showed a decrease compared to the CTRL group (Figure 1I).

**Figure 2:**
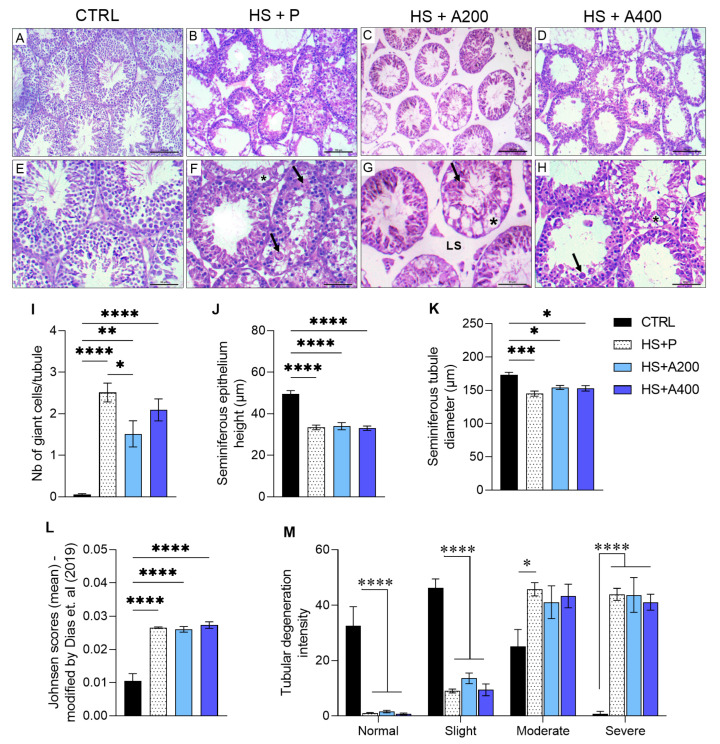
Effects of preventive treatment with alpha-lipoic acid (ALA) on testicular histopathology of mice subjected to scrotal heat shock (HS). Photomicrographs of testis from mice in the CTRL (**A**,**E**), HS + P (**B**,**F**), HS + A200 (**C**,**G**), and HS + A400 (**D**,**H**) groups. The testes exposed to HS showed vacuoles (asterisks) and multinucleated giant cells (arrows). The A200 + HS group (**C**) showed significant lymphatic space (LS). Bars: 100 µm (**A**–**D**); 50 µm (**E**–**H**). (**I**) Mean number of multinucleated giant cells per tubule. (**J**) Height of the seminiferous epithelium. (**K**) Seminiferous tubule diameter. (**L**) Mean Johnsen score following adaptation proposed by Dias et al. [53]. (**M**) Proportions of seminiferous tubule pathologies. Legends: CTRL—control without heat shock (n = 6); HS + P—scrotal heat shock-receiving placebo (n = 10); HS + A200 (n = 7) and HS + A400 (n = 6)—scrotal heat shock and daily doses of ALA; * *p* < 0.05; ** *p* < 0.01; *** *p* < 0.001; **** *p* < 0.0001.

To understand the intensity of these testicular changes, we adopted the adaptation made by Dias et al. [53] in the Johnsen score [54]. We observed a high average score in animals exposed to HS (Figure 2L). In addition, the percentage of seminiferous tubules with mild lesions was lower in HS animals (Figure 2M), but the number of tubules with moderate and severe lesions was higher, except for animals treated with ALA 200 or 400, which showed a number of tubules with moderate lesions comparable to the CTRL group (Figure 2M). Although ALA treatment did not influence most of the morphological and testicular changes, animals that received the 200 mg/kg dose showed a partial improvement in the number of giant cells per tubule (Figure 2I).

### 3.3. Testicular Volumetric Density

In proportion to the volumetric density of tubular components (Table 1), the number of points occupied by seminiferous tubules was lower in the HS + A200 group compared to the other treatments. Regarding the points counted for the seminiferous epithelium and tunica propria, the percentage was significantly reduced for all groups when compared to the CTRL group and even more intensely in the HS + A200 group. The percentage occupied by the lumen was higher in the group without ALA prevention, as well as in 400 mg/kg of ALA, being similar between the HS + A200 and CTRL groups. The total intertubular volume in animals subjected to HS, however, was not re-established by treatment with ALA, and the number of lymphatic spaces was approximately twice as high in animals in the HS + A200 group.

### 3.4. Sperm Kinematics and Morphology

**Figure 3:**
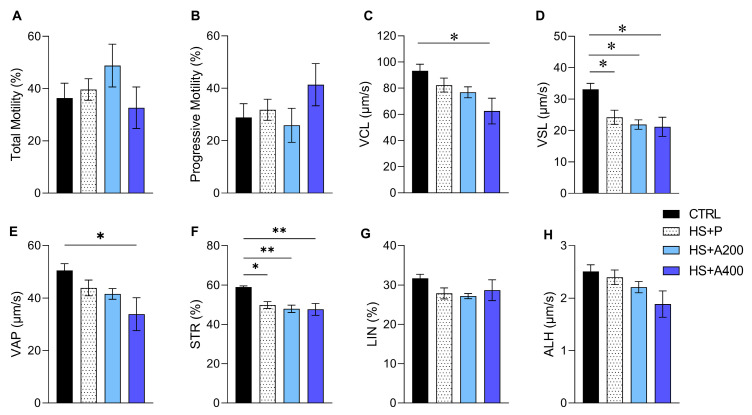
Effects of preventive treatment with alpha-lipoic acid (ALA) on sperm kinematics of mice subjected to scrotal heat shock (HS). (**A**) Total motility (%). (**B**) Progressive motility (%). (**C**) Curvilinear velocity (VCL; μm/s). (**D**) Linear progressive velocity (VSL; μm/s). (**E**) Average path velocity (VAP; μm/s). (**F**) Straightness (STR; %). (**G**) Linearity (LIN; %). (**H**) Amplitude of lateral head displacement (ALH; um/s). Legends: CTRL—control without heat shock (n = 6); HS + P—scrotal heat shock-receiving placebo (n = 10); HS + A200 (n = 7) and HS + A400 (n = 6)—scrotal heat shock and daily doses of ALA; * *p* < 0.05; ** *p* < 0.01.

In the sperm kinematic parameters, we observed that all groups subjected to HS showed reduced VSL (Figure 3D) and STR (Figure 3F) when compared to the CTRL group. In addition, there was a reduction in VCL and VAP in animals that received 400 mg/kg of ALA compared to the CTRL (Figure 3C,E). On the other hand, the percentages for total and progressive motility (Figure 3A,B), LIN (Figure 3G), and ALH (Figure 3H) were not different between groups.

**Figure 4:**
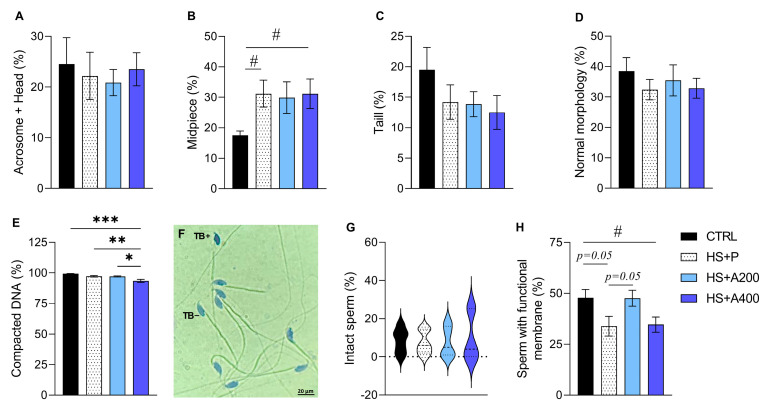
Effects of preventive treatment with alpha-lipoic acid (ALA) on morphology, cytoplasmic membrane integrity, and DNA compaction of mice subjected to scrotal heat shock (HS). (**A**–**C**) Total percentage of head and acrosome defects (**A**), midpiece (**B**), and tail (**C**). (**D**) Sperm with normal morphology. (**E**) Percentage of sperm with compacted chromatin. (**F**) Representative photographs of sperm with (TB+) and without (TB−) penetration (TB−) of toluidine blue (TB) dye. (**G**) Percentage of sperm with intact plasma membrane (CFDA+/IP−). (**H**) Percentage of sperm reactive to HOST. Legends: CTRL—control without heat shock (n = 6); HS + P—scrotal heat shock-receiving placebo (n = 10); HS + A200 (n = 7) and HS + A400 (n = 6)—scrotal heat shock and daily doses of ALA; */#*p* < 0.05; ** *p* < 0.01; *** *p* < 0.001; # versus CTRL group (Student *t* test).

To evaluate the impact of HS and ALA supplementation on sperm morphology and membrane integrity, we analyzed the main structural and functional parameters (Figure 4). Animals in the HS + P group showed an increase in the number of morphological defects in the midpiece when compared to the CRTL group (Figure 4B). For these parameters, only a partial improvement was observed after exposure to 200 mg/kg ALA (Figure 4B,H). For other parameters, such as morphological defects of the head and acrosome (Figure 4A), the tail (Figure 4C), and sperm with a normal morphology (Figure 4D), no statistical differences were observed between treatments. When the types of defects were analyzed separately, we noticed that the animals in the HS + P group exhibited a significant increase in the number of cytoplasmic droplets (Table A1). Although the animals receiving ALA also had high means, this was not statistically different from the CTRL animals (Table A1), demonstrating a partial effect.

### 3.5. Structural and Functional Integrity of Membranes, and Sperm DNA Compaction

To verify the integrity of the cytoplasmic and acrosomal membrane, we submitted the sperm to two fluorescent probes, CFDA and PI. The results of the preventive experiment showed that there were no differences in the percentage of intact (Figure 4G) sperm between the groups. Likewise, the HS + P animals exhibited a trend of a lower number of functional cells in the HOST (Figure 4H), and treatment with ALA 400 mg/kg reduced these values compared to the CTRL group (Figure 4H). Interestingly, treated animals exposed to the 200 mg/kg dose showed a tendency for a higher number of functional cells than the HS + P group, with a number similar to the CTRL group (Figure 4H). Regarding DNA compaction, a lower percentage of cells with more stained nuclei was observed in the HS + A400 group compared to the other groups (Figure 4E,F).

### 3.6. Immunostaining of SOD1 in the Testis

**Figure 5:**
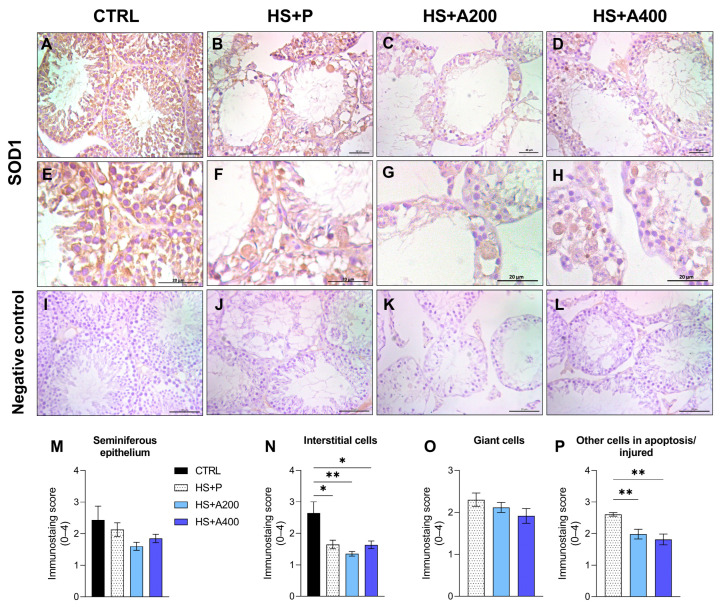
Effects of preventive alpha-lipoic acid (ALA) treatment on testicular superoxide dismutase (SOD1) immunostaining of mice subjected to scrotal heat shock (HS). (**A**–**H**) Photomicrographs of SOD immunostaining in the testes of mice in the CTRL (**A**,**E**), HS + P (**B**,**F**), HS + A200 (**C**,**G**), and HS + A400 (**D**,**H**) groups. (**I**–**L**) Photomicrographs of negative controls. (**M**–**P**) Staining intensity scores in the seminiferous epithelium (**M**), interstitial cells (**N**), giant cells (**O**), and other apoptotic/injured cells (**P**). Bar: 20 μm. Legends: CTRL—control without heat shock (n = 6); HS + P—scrotal heat shock-receiving placebo (n = 10); HS + A200 (n = 7) and HS + A400 (n = 6)—scrotal heat shock and daily doses of ALA; * *p* < 0.05; ** *p* < 0.01.

In order to confirm the effects of HS and ALA treatment on the testicular antioxidant function of mice, in this study, we evaluated the staining of SOD1, the main enzyme involved in the antioxidant response [56]. Intense SOD1 staining was observed in the cell cytoplasm of all testicular components in control mice (Figure 5A,E), a pattern similar to that observed in healthy rats [57]. In animals subjected to HS without ALA treatment, a slightly lower intensity was observed, although it was only significant for interstitial cells (Figure 5N). This pattern, although slightly weaker in animals treated with ALA, did not differ between HS groups (Figure 5C,D,G,H).

Giant cells, when present, exhibited moderate to intense staining, but with no differences between HS groups (Figure 5F,G,H,O). On the other hand, the HS group exhibited more intense staining in other injured cells when compared to treatments with ALA200 or ALA400 (Figure 5F,G,H,P). Figure 5I–L represents the negative controls with replacement of the primary antibody by PBS. Although it was not possible to quantify the pixel area of these images due to the extent of injury in HS animals, there is clear evidence of reduced SOD1 expression associated with testicular HS, but without an influence of ALA treatment in our model.

## 4. Discussion

Testicular dysfunction induced by heat shock (HS) poses a significant challenge to male fertility, compromising cellular parameters essential to sperm quality. Although alpha-lipoic acid (ALA) has been widely studied as a therapeutic agent in various models of testicular dysfunction [32,33,34,35,58,59], its effectiveness in preventing such damage remains unknown. In this study, we investigated for the first time the effects of preventive ALA administration on testicular protection against HS-induced injury in mice. The 30-day administration period was selected to simulate a realistic preventive approach and ensure sufficient time for ALA to exert systemic antioxidant priming. This duration is consistent with previous murine studies using similar or longer treatment periods at comparable doses, which demonstrated efficacy and tolerability [39,40,41,42,43]. Despite its well-known antioxidant properties, our findings indicate that ALA, regardless of the administered dose, had minimal influence on most of the observed deleterious effects, suggesting that, in this model, its prophylactic application may be insufficient to prevent testicular damage caused by HS.

Scrotal HS exposure resulted in significant reductions in body weight and the relative mass of the testes, except in animals treated with 200 mg/kg of ALA, compared to the non-exposed control group. These effects have been widely documented in various chronic heat stress models, including mice [60,61,62] and rats [7,63,64]. The marked reduction in testicular mass following HS exposure reinforces previous findings linking thermal stress to spermatogenic dysfunction. As reviewed by Durairajanayagam et al. [5], this dysfunction is largely attributed to germ cell apoptosis and autophagy, as well as imbalances in testicular homeostasis. Interestingly, animals treated with 200 mg/kg of ALA showed no change in relative testicular mass, which may indicate a role for ALA in preserving testicular size in relation to body weight.

Histomorphometric and histopathological analyses revealed a significant decrease in tubular diameter and seminiferous epithelium height in groups exposed to HS. Moreover, these animals presented severe tubular degeneration, validating the effectiveness of the experimental HS model. The presence of multinucleated giant cells, Sertoli-only tubules, and even complete germ cell loss illustrates the extent of the cellular damage [54]. The testicular changes observed in this study are comparable to those described in models of disrupted thermoregulation [65] and chronic inflammatory processes [66]. In all of these conditions, an increased intratesticular temperature disrupts homeostasis, promoting mitochondrial dysfunction and elevated oxidative stress, leading to progressive degeneration [66,67]. If hyperthermia is not corrected, spermatogenesis becomes severely compromised, resulting in a significant decline in male fertility. Preventive treatment with ALA at 200 mg/kg attenuated the formation of multinucleated giant cells, suggesting a reduction in uncontrolled cell death, as these cells result from meiotic disruption in germ cells and subsequent mutational events involving damage to intercellular bridges due to a failure of Sertoli cytoskeletal support, migrating into the lumen if not eliminated by phagocytosis [68,69]. Furthermore, regardless of the dose, ALA normalized the quantity of moderate lesions, indicating a partial effect in preserving the tubular architecture.

An interesting finding was the increased interstitial space in the group treated with 200 mg/kg of ALA, possibly associated with vascular dysfunction and interstitial fluid accumulation in testicular tissue, which may have contributed to reduced testosterone levels in this group. During necropsy, testes from placebo and 200 mg/kg ALA-treated animals were soft to palpation and exhibited scrotal edema, suggesting structural compromise of the testicular tissue. Histological assessment of volumetric density revealed that the 200 mg/kg group had a lower percentage of seminiferous tubules, epithelium, and Leydig cells, which may be explained by increased lymphatic intertubular space, indicating edema [70]. These structural alterations may compromise Leydig cell functionality and, consequently, hormonal production. This hormonal reduction is especially noteworthy, as testosterone plays a central role in maintaining spermatogenesis and testicular homeostasis. Although ALA is widely recognized for its antioxidant and cytoprotective effects, the combination of high-dose administration and prolonged exposure may, in certain physiological conditions, lead to paradoxical impacts on steroidogenic function.

The observed edema and decrease in Leydig cell area might suggest impaired vascular exchange or inflammatory signaling that disrupts testosterone biosynthesis. Additionally, despite evidence from other studies showing that ALA may restore testosterone levels under toxic or oxidative conditions in rats [71,72], our findings contrast with that trend, suggesting that in the context of prophylactic use in thermally stressed murine models, this compound might exert different endocrine effects. Future research should include hormonal profiling beyond serum testosterone—such as LH and FSH levels—as well as analysis of genes involved in steroidogenesis, to clarify whether ALA affects the hypothalamic–pituitary–gonadal axis directly or indirectly.

Regarding sperm parameters, few effects of scrotal HS were observed. These included reductions in VSL (μm/s), STR (%), and the number of spermatozoa with functional plasma membranes, in addition to an increase in morphological defects in the midpiece. It is well established that the most pronounced impacts on spermatogenesis and sperm quality tend to emerge after 7 to 14 days of exposure, rather than within short periods such as 48 h. For instance, Dang-Cong and Nguyen-Thanh [73], in a study on mice, demonstrated that seminiferous epithelium degeneration and significant germ cell loss are progressive and become more pronounced over time. Similarly, Abd El-Emam et al. [74], studying rats, reported that sperm motility and the sperm count showed a dramatic decline after 14 days, whereas shorter-term evaluations indicated less expressive changes. Additionally, Zhao et al. [75] observed that in mice, reductions in motility and sperm concentration were more evident after seven days, whereas 24 h assessments showed milder effects. In a bovine study, Capela et al. [76] reported that ROS elevation and decreased sperm motility were progressive, becoming more evident after 14 and 21 days. Another relevant factor is the cellular response to thermal stress. Afshar et al. [77] demonstrated that HSP70 and HSP90 heat shock protein expression in mice increased significantly after seven days, suggesting a cellular adaptation mechanism that may influence sperm recovery. Ziaeipour et al. [78], in a study with mice, showed that 35-day HS exposure induced azoospermia and severe testicular damage, reinforcing that HS effects can intensify over time.

Nonetheless, early evaluations may still capture direct effects on epididymal spermatozoa, especially under intense thermal protocols such as the one used in the present study (43 °C for 20 min). Prior studies have demonstrated that scrotal HS exposure can lead to increased DNA fragmentation, oxidative stress, and mitochondrial dysfunction within 24 to 48 h [6,79,80]. Thus, although the full extent of damage may emerge over longer periods through spermatogenesis disruption, early changes in sperm motility and morphology may reflect immediate stress responses in sperm cells already present in the epididymis. Future studies should therefore incorporate longer post-exposure intervals (e.g., 7 to 14 days) to better characterize the progressive effects of heat stress and the temporal dynamics of ALA’s potential protective role.

Preventive ALA treatment had limited impact on sperm alterations induced by scrotal HS. However, the 200 mg/kg dose appeared more beneficial, as it did not differ from the control group regarding midpiece defects and sperm membrane functionality. In contrast, animals treated with 400 mg/kg of ALA exhibited a lower VCL (μm/s) and reduced numbers of spermatozoa with compacted chromatin—a finding consistent with DNA strand breaks induced by HS [79]. Additionally, like the HS-only group, they showed decreased numbers of sperm with functional membranes. These findings suggest that the higher ALA dose may have been ineffective in preserving the nuclear structure and membrane integrity, differing from the effect observed at 200 mg/kg.

SOD1 immunolabeling assessment revealed a reduced expression in testicular tissue from HS-exposed animals, consistent with other studies evaluating antioxidant enzyme expression following HS [38,75,81]. Nonetheless, the 30-day pretreatment with ALA at either dose did not influence this outcome. Therefore, although ALA is recognized for its antioxidant potential, as demonstrated in poultry models [38], its preventive application may not be sufficient to fully preserve testicular antioxidant enzyme activity under HS conditions. Given the scope and technical constraints of the current study, SOD1 was selected as a viable marker due to its clear immunoreactivity and functional relevance in testicular oxidative responses. A significant limitation of this study was the lack of evaluation of other endogenous antioxidants, such as catalase and glutathione peroxidase, which could provide further insights into the cellular mechanisms involved in testicular responses to HS, as well as direct biochemical markers (e.g., malondialdehyde [MDA], reduced/oxidized glutathione ratio [GSH/GSSG]) and gene expression analysis. These complementary approaches are necessary in future studies to fully characterize the oxidative stress pathway and the antioxidant mechanisms modulated by ALA in heat-stressed testicular tissue.

Despite evidence suggesting a potential preventive effect of supplementation with 400 mg/kg/day of ALA in mice, it is essential to emphasize that both tested doses (200 and 400 mg/kg/day) resulted in a significant mortality rate, around 30–40% throughout the experimental period. This finding raises important concerns regarding the safety of prolonged ALA administration in murine models. The literature indicates that mice are considerably more susceptible to the toxic effects of alpha-lipoic acid than rats—whose average oral LD_50_ is approximately 1130 mg/kg, while in mice it is around 502 mg/kg [82,83]. The animals that died presented clinical signs compatible with systemic toxicity, such as weight loss, lethargy, dull fur, and respiratory distress. Previous studies have shown that high doses of ALA in mice can induce hepatic, neurological, and metabolic adverse effects, even in short-term protocols [84]. Furthermore, there is evidence that chronic administration of ALA in mice, even at moderate doses (20 mg/kg), can lead to significant hepatic alterations, such as increased liver enzymes and inflammation [83].

Notably, studies employing high doses of ALA in mice have reported divergent outcomes: while some protocols (e.g., ~300–500 mg/kg/day for 8 to 20 weeks) demonstrated tolerability without mortality [40,41,42], others, such as Farr et al. [84], described a reduced lifespan even with 100 mg/kg/day in aging-prone mice. These findings suggest that the strain susceptibility, route of administration, treatment duration, and physiological condition may critically influence ALA’s safety profile. While studies in rats report increased liver and kidney weights following oral ALA administration without significant histopathological findings [82], these data cannot be directly extrapolated to mice due to their higher sensitivity. Therefore, the findings of the present study underscore the need for detailed toxicological investigations of ALA supplementation in murine models, particularly when administered chronically or at doses approaching known toxicity thresholds.

## 5. Conclusions

Our study demonstrates that, after a 30-day pretreatment period and subsequent evaluation within a 48 h post-HS interval, ALA administration was ineffective in preventing testicular and sperm damage induced under the specific experimental conditions employed. Our findings further showed that higher doses of ALA led to significant mortality, reaching 30% and 40% at doses of 200 mg/kg and 400 mg/kg, respectively. Moreover, only two specific sperm parameters, VCL and DNA compaction, were negatively impacted at the highest dose tested (400 mg/kg). These results highlight the complex interaction of ALA with testicular metabolism and provide critical findings regarding its effects after acute heat injury, particularly emphasizing critical safety concerns at high doses.

## Figures and Tables

**Table 1:** Volumetric density (%) of testis components from adult Swiss mice treated with alpha-lipoic acid (ALA) 30 days before acute testicular degeneration by heat shock (HS) (mean ± SEM).

	CTRL	HS + P	HS + A200	HS + A400	*p*-Value (ANOVA)
Seminiferous tubules	85.48 ± 1.19 ^a^	79.11 ± 3.16 ^a^	59.61 ± 2.36 ^b^	78.87 ± 1.81 ^a^	<0.0001
Tunica propria	9.16 ± 0.48 ^a^	5.28 ± 0.90 ^b^	5.09 ± 0.48 ^b^	4.23 ± 0.54 ^b^	<0.0001
Seminiferous epithelium	60.01 ± 2.42 ^a^	46.18 ± 2.79 ^b^	36.14 ± 1.42 ^c^	46.92 ± 2.56 ^b^	<0.0001
Lumen	16.31 ± 1.32 ^b^	27.64 ± 1.38 ^a^	18.39 ± 2.10 ^b^	27.71 ± 1.70 ^a^	<0.001
Intertubular compartment	14.52 ± 1.19 ^b^	20.89 ± 3.16 ^b^	40.39 ± 2.63 ^a^	21.13 ± 1.81 ^b^	<0.0001
Leydig cell	3.60 ± 0.37 ^ab^	4.18 ± 0.29 ^a^	2.77 ± 0.18 ^b^	3.80 ± 0.46 ^ab^	0.0341
Blood vessels	1.02 ± 0.23 ^a^	1.12 ± 0.19 ^a^	0.88 ± 0.19 ^a^	1.46 ± 0.10 ^a^	0.1788
Lymphatic space	6.48 ± 1.61 ^b^	10.76 ± 2.20 ^b^	33.75 ± 2.35 ^a^	12.26 ± 1.83 ^b^	<0.0001
Connective tissue	3.43 ± 0.75 ^a^	4.83 ± 1.23 ^a^	2.98 ± 0.41 ^a^	3.61 ± 0.32 ^a^	0.5268

CTRL—control without heat shock (n = 6); HS + P—scrotal heat shock-receiving placebo (n = 10); HS + A200 (n = 7) and HS + A400 (n = 6)—scrotal heat shock and daily doses of ALA. Different letters indicate significant differences (*p* < 0.05) among groups.

## Data Availability

The data supporting the findings of this study are available from the corresponding authors, L.C.S. and P.P.d.N.S., upon reasonable request.

## Abbreviations

The following abbreviations are used in this manuscript:

ALA	Alpha-lipoic acid
HS	Heat shock
CTRL	Control group
VSL	Linear progressive velocity
STR	Straightness
SOD	Superoxide dismutase
SOD1	Superoxide dismutase 1
ROS	Reactive Oxygen Species
CAT	Catalase
GPx	Glutathione peroxidase
DHLA	Dihydrolipoic acid
LPS	Lipopolysaccharide
LaBIO	Laboratory of Animal Breeding, Maintenance, and Experimentation
UESC	Santa Cruz State University
ARRIVE	Animal Research: Reporting of In Vivo Experiments
CEUA	Ethics Committee on Animal Use
I.P.	Intraperitoneal
TM	Total motility
PM	Progressive motility
VCL	Curvilinear velocity
VAP	Average path velocity
LIN	Linearity
ALH	Amplitude of lateral head displacement
HOST	Hypoosmotic test
CFDA	Carboxyfluorescein diacetate
PI	Propidium iodide
SCA	Sperm class analyzer
RIA	Radioimmunoassay
IHC	Immunohistochemistry
PBS	Phosphate-buffered saline
H&E	Hematoxylin and Eosin
DM2500	Leica DM2500 Microscope
DFC 295	Leica DFC 295 Digital Camera
ANOVA	Analysis of variance
SEM	Standard error of the mean
Shapiro-Wilk	Shapiro–Wilk normality test
Kruskal-Wallis	Kruskal–Wallis test
Dunn	Dunn multiple comparison test
GI	Glandular index
TB	Toluidine blue
TB+	Toluidine blue positive
TB−	Toluidine blue negative
HSP70	Heat shock protein 70
HSP90	Heat shock protein 90
MDA	Malondialdehyde
GSH/GSSG	Reduced/oxidized glutathione ratio

## Appendix A

Different types of defects in sperm morphology are shown in Table A1.

**Table A1:** Effects of preventive treatment with alpha-lipoic acid (ALA) on sperm morphology of mice subjected to scrotal heat shock (HS).

	CONTROL	HS + P	HS + A200	HS + A400	ANOVA	
					*F*-Value	*p*-Value
Acrosome defects	2.52 ± 0.90	3.60 ± 1.33	6.57 ± 1.85	4.00 ± 1.29	1.32	0.290
Head defects	10.83 ± 2.52	12.80 ± 2.47	10.57 ± 1.88	12.17 ± 2.00	0.22	0.880
Isolated head	8.66 ± 3.36	3.55 ± 0.55	3.71 ± 1.30	7.33 ± 2.37	1.81	0.171
Tail	0.50 ± 0.22	1.10 ± 0.45	0.71 ± 0.28	1.60 ± 1.16	0.61	0.610
Cytoplasmic droplet	8.50 ± 1.43 b	21.00 ± 3.26 a	15.00 ± 3.42 ab	21.00 ± 3.76 ab	3.40	0.035
Midpiece	9.00 ± 0.85	13.11 ± 3.33	10.71 ± 2.90	12.67 ± 2.41	0.43	0.731
Main piece	17.50 ± 3.73	12.60 ± 2.87	12.57 ± 2.33	8.33 ± 1.83	1.41	0.262
End piece	0.00 ± 0.00	0.50 ± 0.26	0.57 ± 0.42	0.66 ± 0.33	0.64	0.593

Control—no heat shock; HS + P—heat shock + placebo; HS + A200 and HS + A400—heat shock and daily doses of ALA. Different letters indicate significant differences (*p* < 0.05) among groups. Data shown as mean + standard error of the mean (SEM).
